# Intra and Inter-Population Morphological Variation of Shape and Size of the Chilean Magnificent Beetle, *Ceroglossus chilensis* in the Baker River Basin, Chilean Patagonia

**DOI:** 10.1673/031.011.9401

**Published:** 2011-07-26

**Authors:** Hugo A. Benítez, Raúl Briones, Viviane Jerez

**Affiliations:** ^1^ Departamento de Zoología, Facultad de Ciencias Naturales y Oceanográficas, Universidad de Concepción, Concepción, Casilla: 160-C - 4070386, Chile; ^2^ Instituto de Alta Investigación, Universidad de Tarapacá, Casilla 7-D Arica Chile; ^3^ Departamento de Ecología y Biodiversidad, Facultad de Ecología y Recursos Naturales, Universidad Andrés Bello, República 470, Santiago, Chile

**Keywords:** geometric morphometric, habitat fragmentation, isolation, interpopulation, mature forest, Second-growth for dimorphism

## Abstract

The alteration of habitat generates different degrees of stress in insects. It has been suggested that the degrees of phenotypic disturbances reflect the ability of an individual to overcome the effects of stress. The Baker River Basin in the Aysén Region, Chilean Patagonia has a very fragmented landscape, due to the destruction of the native forest and the use of land for agriculture and animal husbandry. This alteration should generate different degrees of disturbances in the insect communities, whose effects may be quantified by geometric morphometric tools. We analyzed morphological differences in 244 males and 133 females of the the Chilean magnificent beetle, *Ceroglossus chilensis* (Eschscholtz) (Coleoptera: Carabidae) collected in January, 2007, in mixed forests of *Nothofagus dombeyi* Mirbel (Ørsted) (Fagales: Nothofagaceae) and *N. nitida* Hofmus and in Second-growth forest of *N. pumilio* (Poepp. & Endl.) Krasser. Males were generally wider in the pronotum, while females had wider abdominal sternites. Although there were significant differences in shape and size between mature forests and second-growth forest, these were less significant among the sites within each type of vegetal formation. Individuals had more shape variations in the mature forest. We suggest that differences in shape are due at least in part to the isolation of the habitat. The differences found between sexes raises the question of how morphological variations and sexual dimorphism may be affected spatially by natural selection.

## Introduction

The ecological region of the temperate forest of southern Chile has a long history of geographic isolation, generating a narrow distribution range and high levels of endemism in its biota, which has been unaffected by anthropogenic disturbance since the Quaternary. Unfortunately, for more than 70 years the Aysén Region has been subject to several events that have endangered its biodiversity, especially extensive burning of forests, use of land for agriculture and animal husbandry, and, more recently, colonization (Espinoza et al. 1994, Quintanilla 2008). As a result, the landscape, particularly in the southern part of the region, has been dramatically modified from its original state.

Floate and Fox (2000) and Piscart et al. (2005) have shown that the degrees of phenotypic disturbances reflect the ability of an individual to overcome the effects of stress, suggesting that more symmetrical individuals would have a greater survival probability than those with high levels of asymmetry. However, because of adaption over time to a specific environment, environmental pressures and geographic distances affect geographic microenvironments locally and thus their associated flora and fauna (Alibert et al. 2001; Cepeda-Pizarro et al. 2003; Benítez et al. 2008). There is evidence that temperatures, adverse nutritional stress, chemical presence, density of population and many other factors causing stress during development can lead to increased fluctuating asymmetry (e.g. Rettig et al. 1997; Benitez et al. 2008, Henríquez et al. 2009; Benitez et al. 2010b). Therefore, it is expected that when environmental conditions change, organisms and populations should adapt to the new conditions (Clarke 1993). As a consequence, the generation of symmetric phenotypes is conditioned by the dampening of the phenotype in response to the disturbances that occur during morphogenesis (Labrie et al. 2003; Leamy and Klingenberg 2005).

*Ceroglossus* is a genus of carabid beetles endemic to the forests of southern South America that include eight species: the Chilean magnificent beetle, *C. chilensis* (Eschscholtz) (Coleoptera: Carabidae), *C. darwini* (Hope), *C. speciosus* Gerstaecker, *C. magellanicus* Géhin, *C. buqueti* (Laporte), *C. suturalis* (Fabricius), *C. ochsenii* (Germain), and *C. guerini* (Germain), all of diurnal habit and predators on smaller organisms.

A marked chromatic polymorphism has been found in populations of *Ceroglossus*, which may be associated with environmental differences like temperature and humidity (Jiroux 2006). This has been corroborated in *C. chilensis* with DNA analysis (Okamoto et al. 2001).

*Dense activity: temporal and spatial variations of relative abundance, determined by the effect they have some atmospheric variables on activity and the presence of barriers that limit their free movement

*C.* *chilensis* has 26 subspecies, distributed from the Maule Region and the extreme south of the Aysén Region; it is also present in Argentina, and is the southernmost species and the one with the widest distribution in Chile. It prefers more xeric habitats and is more tolerant of arid conditions than its congeners. It is not known if its size, which is relatively large for a carabid, is related to its ability to resist the aridity of the environment (Jiroux 2006). There is evidence that the development and environmental instability of *C. chilensis*, a species with a high activity density, is affected by the modified environment versus the natural environment (Briones and Jerez 2007; Benitez et al. 2008, Henríquez et al. 2009).

The Baker River Basin has the largest water volume of any river in Chile; it is 200 km long, drains an area of 26,487 km^2^ and has its origin in Lake Bertrand. In the Baker River Basin, *C. chilensis* is found in mixed mature evergreen forests containing *Nothofagus dombeyi* Mirbel (Ørsted) (Fagales: Nothofagaceae) and *N. nitida* Hofmus, and in second-growth forests containing *N. pumilio* (Poeppig et Endl.) Krasser (Rodriguez et al. 2008). There is an approximate distance of 85 km isolating these two habitats (Benitez 2008), an area that is populated by diverse vegetation types from anthropogenic intervention, exotic plantations, grasslands, antarctic beech forests of *Nothofagus antartica* (G. Forster) Oerst, and shrublands with *Embothrium coccineum* J.R. et G. Forster (Proteales: Proteaceae). Therefore, since the same insect species exist in these two geographically isolated environments, we may expect to find different morphotypes as a response of this species to the different environmental disturbances occurring particularly in each of these sites.

**Table 1.**
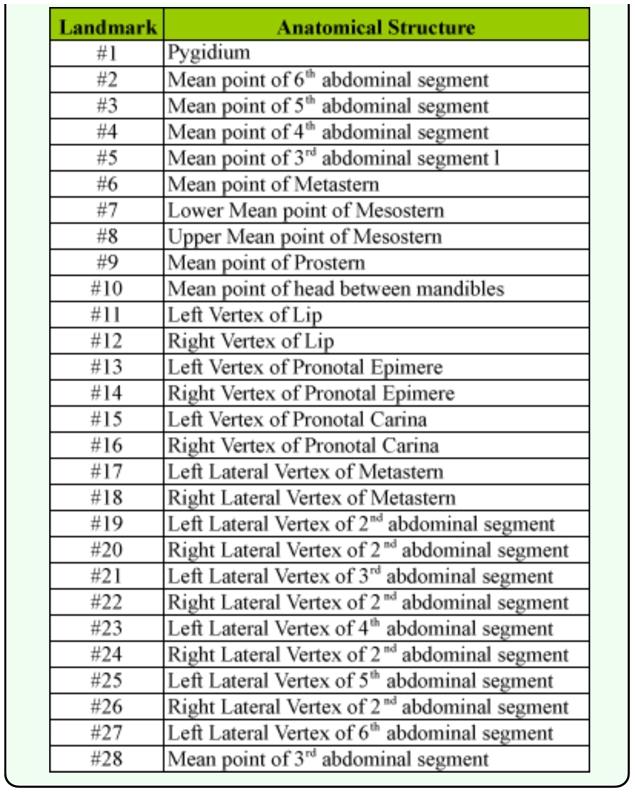
Anatomical description of 28 external landmarks in *Ceroglossus* *chilensis* body shape.

The objective of this study was to evaluate the morphological differentiation and the sexual dimorphism within and between populations of *C. chilensis* in different and isolated geographic areas of the Baker River Basin, using geometric morphometric.

## Materials and Methods

### Data acquisition

**Figure 1.**
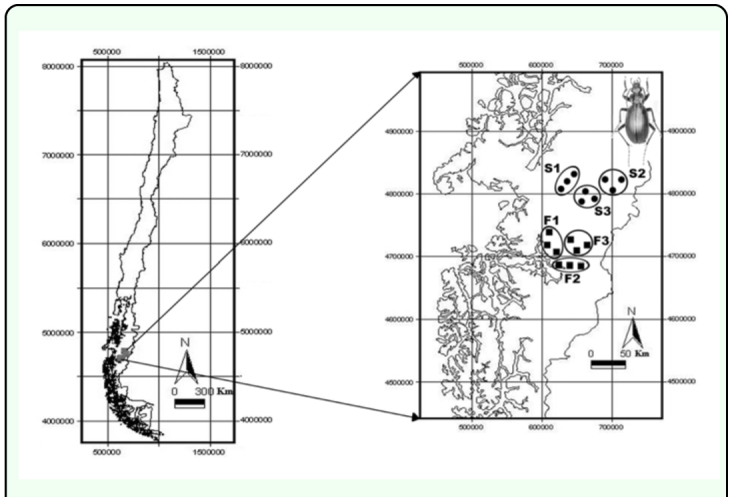
Map of Chile and the Aysén Region, indicating the study area and the sampling sites. Circles: Second-Growth Stand, Squares: Forest. High quality figures are available online.

Eighteen sampling sites were selected, separated into 9 sites in mature forests (F1, F2 and F3) and 9 sites in second-growth forests (S1, S2 and S3). Figure 1 shows their spatial location. During January 2007, we installed in each site 12 pitfall traps, separated by approximately 5 m, for 3 days and 3 nights. The sex of individuals was determined under an optical microscope, based on the presence of antennal careens (Benítez et al. 2010a).

To determine whether our results in diversity are the product of the distance between the sample and not replicas by forest type, we performed the Mantel test using Poptools version 2.6.6 (Hood 2005). We used the difference of similarity matrices of high activity density, regarding the geographical distances between forest types.

### Data analyses

**Figure 2.**
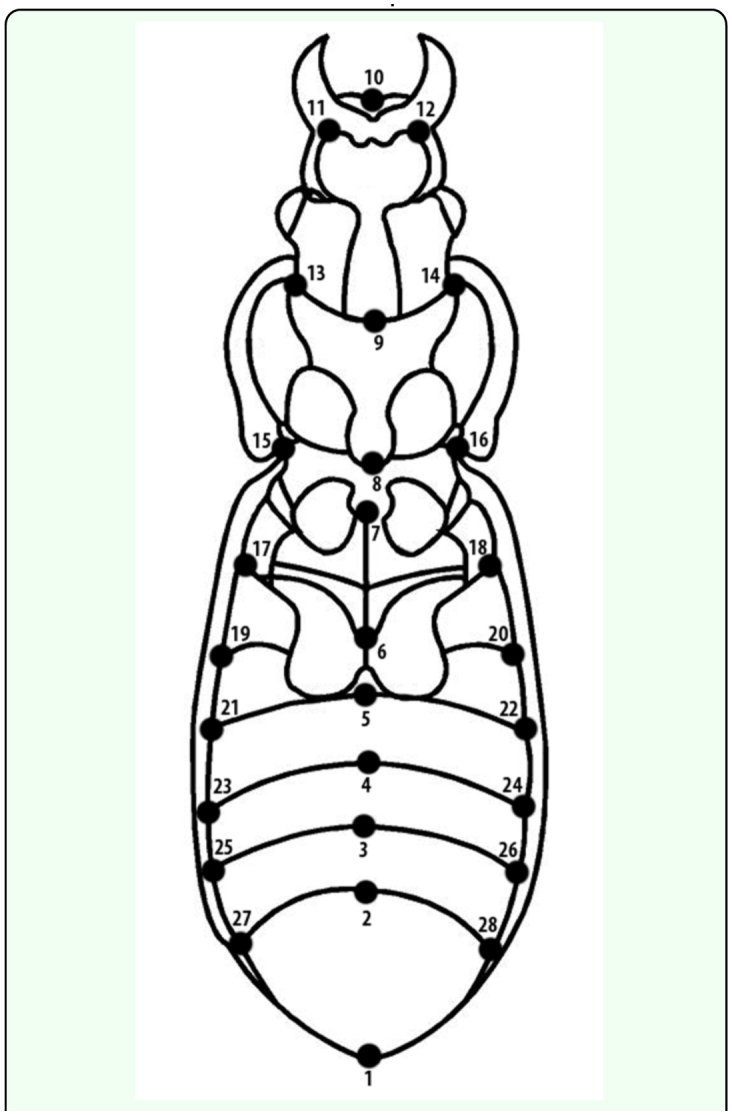
*Ceroglossus chilensis.* Indication of 28 landmarks in the ventral view. High quality figures are available online.

The geometric analysis considered exclusively variations in shape, and was performed using a photograph in ventral view of males and females with an Olympus X-715 digital camera (www.olympus.com) (Bookstein 1996). Using the methodology of Alibert et al. (2001), 28 landmarks were digitalized (Figure 2, Table 1). The selection of morphological landmarks was based on external anatomy and homologous characters using type 1 landmarks (anatomical) (Zelditch et al. 2004). These were digitalized for each individual using the program TpsDig 2.12 (Rohlf 2001). The XY coordinates of homologous landmarks, were aligned and superimposed with the program TpsSuper 1.06 (Rohlf 2003b), using the method of minimum least squares based on the *Generalized Procrustes Analysis* (Rohlf and Slice 1990). The shape variables of aligned individuals were obtained with the program Tps Relw 1.42 (Rohlf 2003a), which creates an interpolation function that projects the data in a Euclidean plane. A principal components analysis (*relative warp analysis*) was performed with this same program. Some variations in body size were analyzed by means of centroid size calculation using landmarks 1 to 10.

The TPS Thin-Splate software was used to evaluate local shape variation by calculating λ parameters. In this analysis, values near 1 determine local variations and values near 0 mean global variation of shape. The main purpose of this type of analysis on non-uniform changes is to show how much localized or generalized these shape variations may be.

### Statistical analyses

To estimate intrapopulation differences, we performed 2-way factorial ANOVAs using locality and sex as factors. To estimate differences among populations, as well as the 2-way factorial ANOVA, we evaluated the differences using all variables with a MANOVA analysis. All these analyses were performed with the program STATISTICA 7.0 (Statsoft 1999).

### Measurement error

To diminish the measurement errors and avoid bias in taking photographs and digitalizing landmarks, we selected a random sub-sample of 180 individuals, took new photographs and made new digitalization of the morphological landmarks. The proportional measurement error compared to the real data had relative contributions of 3.5% (Rw1) and 0.46% (Rw2) for forest and 1.13% (Rw1) and 0.08% (Rw2) for second-growth forest. The percentage of error for both vegetal formations and all sites was less than 4%.

## Results

We collected a total of 477 individuals of *C. chilensis*, 148 males and 140 females in mature forest and 96 males and 93 females in second-growth forest. The high activity density (*sensu* De los Santos et al. 2002) for mature forests indicates 12.8 ind/trap and 3.5 ind/trap for second-growth forest. The Mantel test applied to the data of high activity density, indicated that sampling units are not spatially auto-correlated and are statistically independent. This indicates that there is no significant relationship between the differences of high activity density (*p* = -0.25) of *C. chilensis* and their spatial separation.

To quantify body size, we used centroid size, which is a measure of the spread of landmarks around their centre of gravity (Dryden and Mardia 1998). A multivariate regression for the variable shape and centroid size, plus a generalized Goodall F test (F = 7.8223; df = 52. 2189; *p* < 0.0001) discounted the presence of an allometric factor; there were not large differences between the first axis of the principal components (shape variables and the logarithm of centroid size).

Non-uniform variations (k-3 principal warps, where k is the number of landmarks) were calculated for mature forest (F) and second-growth forest (S). The deformations were calculated with the first and last principal warps, with which the global changes for F had a folding index of λ = 5.2599E-007 and λ = 0.0001855 for local changes, while S had global λ = 6.0965E-007 and local λ = 0.0001819. The global changes were similar for both formations.

### Sex and Locations Differences

The factorial ANOVA in F found significant differences among sampling sites but not between sexes using Rw1 as dependent variable; F = 5.3044; *p* = 0.001. Incorporating the two other relative warps (Rw2 and Rw3), which explained almost all of the variation in shape (86.5%) as dependent variables, we found differences among sites and between sexes F = 11.9966; *p* < 0.001 (locality) and ANOVA F = 6.225; *p* < 0.001 (sex). Similar results were obtained for S; differences among sites were significant for S (ANOVA: F = 24.058; *p* < 0.001), and incorporating the other two relative warps, which as dependent variables explained 88.13% of the variation in shape, there were significant differences both for sex and for locality F = 9.172; *p* < 0.001 (locality) ANOVA F = 9.9208; *p* < 0.001 (sex).

**Figure 3.**
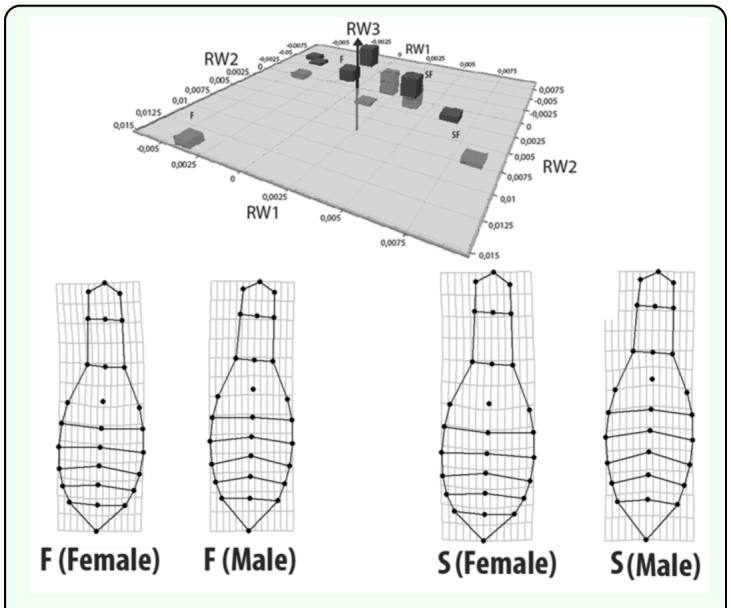
3D bar dispersion plot showing distributions of the shape for males and females in the sampling sites for Mature Forest (F) and Second-growth forest (S). *M (males) * F (females). High quality figures are available online.

**Figure 4.**
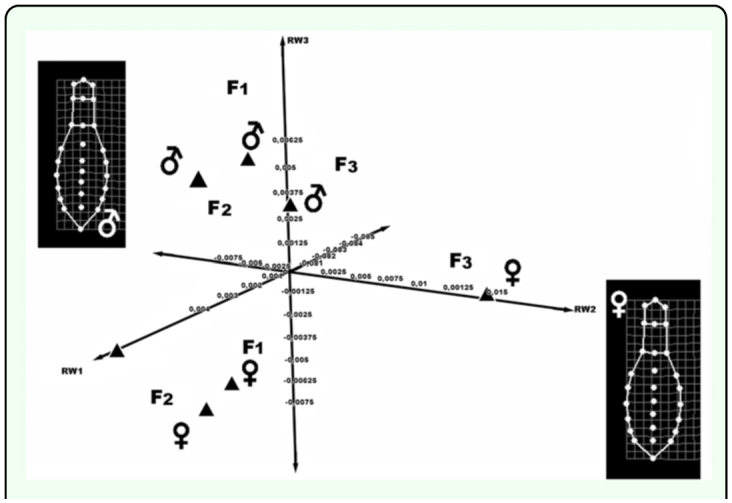
3D dispersion plot showing the relative positions of the different sizes and sexual dimorphism in Mature Forest. The deformation grids indicate a mean shape variable for males and females. High quality figures are available online.

**Figure 5.**
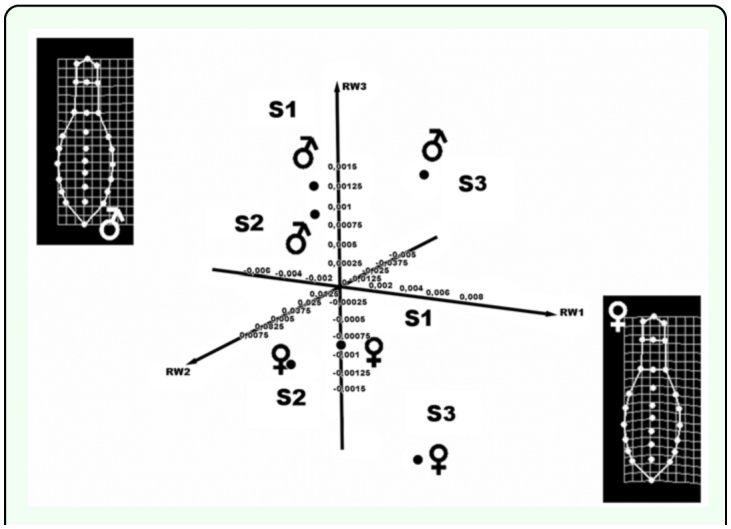
3D dispersion plot showing the relative positions of the different sizes and sexual dimorphism in Second-growth forest. The deformation grids indicate a mean shape variable for males and females. High quality figures are available online.

The morphological variation of individuals in both types of vegetation was estimated by fusing their matrices using TpsUtil 1.4, using the method of minimum mean squares (Generalized Procrustes Analysis) (Rohlf and Slice 1990). Significant differences were found in morphology between vegetation types and between sexes ANOVA: F = 5.6238; *p* < 0.0001. These differences were visualized graphically with a 3D bar plot (Figure 3) that shows the associations among populations and sexes. The difference in size between sexes was significant for both vegetation types. The differences in shape were also significant in both cases; males were generally wider in the area of the pronotum, while females had wider abdominal sternites (ANOVA F = 5.2375; *p* = 0.005) (Figures 4 and 5).

## Discussion and conclusions

The variation in shape of *C. chilensis* was clearly demonstrated by the techniques of geometric morphometric. Although there were differences in shape and size between forests and second-growth forest, these were less different among the sites within each type of vegetal formation. The maximum distance between sites was 85 km (F1 and S3) (Benitez 2008), thus it is feasible that the differences in shape, in addition to being local differences, are due at least in part to the fragmentation of the habitat (Henríquez et al. 2009).

Our results indicate that morphological variations and the variation among sampling sites are due to differences in shape, not to size (Adams and Funk 1997). Alibert et al. (2001) suggested that size variations among populations are necessarily influenced by the environment. However, the disturbances generated by anthropogenic activity have had a historic influence in the Aysén Region, generating a highly heterogeneous vegetation landscape.

The results of this study show that *C. chilensis* has only a small amount of morphological variation among sites of the same vegetation type, but have formed discrete units as a result of isolation between mature forest and second-growth forest.

Although these variations are not visible to humans by ocular inspection, they may be sufficient to produce sexual selection by the insects. The variation in the abdomen was greater in females; this is an essential morphological character which allows a female to produce more eggs and therefore have a greater fecundity and greater fitness (Andersson 1994; Cepeda-Pizarro et al. 1996; Benítez et al. 2010a; Benítez et al. 2010b).

Although there were significant differences in size among populations, we cannot argue that these are only due to sexual dimorphism. It is frequently suggested that size variation of individuals may be strongly dependent upon unfavorable environmental conditions (Adams and Funk 1997; Tatsuta et al. 2001). Thus we conclude that the differences between sexes and among sampling sites are significant for the studied vegetation types. The differences between sexes raise the question of how morphological variation and sexual dimorphism may be affected spatially as a result of natural selection.

## Abbreviations

F,: mature forest;
S,: second-growth forest
